# Demographic and anthropometric characteristics and their effect on the concentration of heavy metals (arsenic, lead, chromium, zinc) in children and adolescents

**DOI:** 10.1016/j.heliyon.2023.e13621

**Published:** 2023-02-09

**Authors:** Majid Hashemi, Saeed Rajabi, Mostafa Eghbalian, Joanna Suliburska, Habibeh Nasab

**Affiliations:** a Environmental Health Engineering Research Center, Kerman University of Medical Sciences, Kerman, Iran; b Department of Environmental Health Engineering, Faculty of Public Health, Kerman University of Medical Sciences, Kerman, Iran; c Student Research Committee, Shiraz University of Medical Sciences, Shiraz, Iran; d Department of Environmental Health Engineering, School of Health, Shiraz University of Medical Sciences, Shiraz, Iran; e Endocrinology and Metabolism Research Center, Institute of Basic and Clinical Physiology Sciences, Kerman University of Medical Sciences Kerman, Iran; f Department of Human Nutrition and Dietetics, Faculty of Food Science and Nutrition, Poznań University of Life Sciences, Poznań, Poland; g Environmental Science and Technology Research Center, Department of Environmental Health Engineering, School of Public Health, Shahid Sadoughi University of Medical Sciences, Yazd, Iran

**Keywords:** Obesity, Body mass index, Heavy metals, Demographic factors, Children, Adolescents

## Abstract

**Background:**

Biomonitoring is a well-established method for assessing people's exposure to contaminants in the environment. Many non-communicable diseases can be prevented or aggravated by physiologically monitoring heavy metals in biological matrices such as urine, evaluating their association with non-communicable diseases, and attempting to limit exposure to them. The focus of this research was to determine the association between potentially toxic elements (PTE) such as arsenic (As), lead (Pb), chromium (Cr), and zinc (Zn) urine concentrations and anthropometric indices and demographic data in children and adolescents aged 6–18 years in Kerman, Iran.

**Methods:**

106 children and adolescents aged 6–18 years in Kerman were randomly selected. A questionnaire was used to acquire demographic information from the participants' parents. Height, weight, and waist circumference (WC) were all assessed, as well as body mass index (BMI) and BMI Z-score. Induced Coupled Plasma Mass Spectrometry (ICP/MS) was used to quantify As, Pb, Cr, and Zn concentrations in participants' urine.

**Results:**

The geometric mean concentrations were As (38.72 ± 39.30), Pb (19.58 ± 22.91), Cr (1.06 ± 0.28), and Zn (344.72 ± 288.16) μg/creatinine. Boys aged 12–18 years old had higher mean concentration of As than boys aged 6–11 years old (p = 0.019) according to two measurement standards, μg/L, and μg/creatinine, whereas girls had no significant difference. In general, there was a strong association between parental education and metal concentrations of As, Pb, and Cr. As, Pb, and Zn (μg/creatinine) had a significant positive association with BMI z-score and BMI. As, Pb, and Zn metals were shown to have a substantial positive association (p < 0.001). There was no evidence of an association between the metals evaluated and WC.

**Conclusions:**

The findings of this study generally showed that there was a significant association between demographic characteristics and exposure to these metals in children and adolescents, indicating that these people were exposed to these metals, which can harm their health. As a result, the pathways of exposure to metals must be limited.

## Graphical abstract

**Figure:**
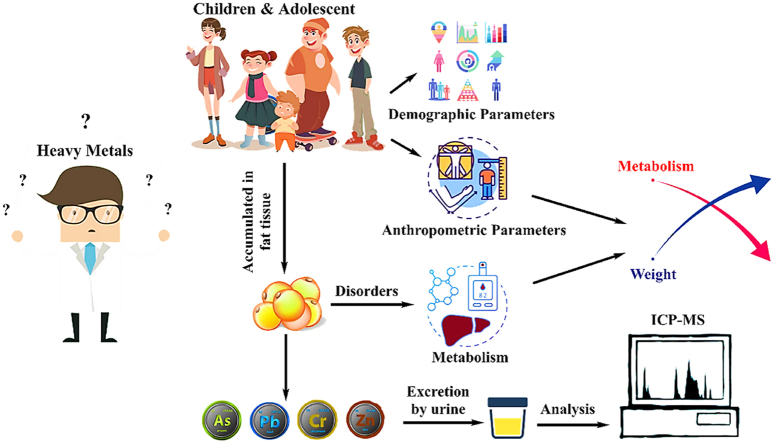

## 1 Introduction

Metals are found in nature and are also employed in industry, medicine, and other applications. As a result, humans are exposed to metals through water, air, soil, food, and consumer products, either naturally or artificially [1]. Heavy metals, also known as potentially toxic elements (PTEs), are a group of chemical pollutants found in the environment that may enter the body through a variety of routes, including skin contact, inhalation, and ingestion [2,3].

Exposure to PTE can have biological consequences in children, mostly attributable to oxidative damage. PTE accumulation in the body is associated with a multitude of pathological diseases in children, including stunted growth, obesity, and cognitive, pulmonary, and neurological disorders [2]. Due to their extended half-lives, heavy metals may accumulate in the human body for decades after being ingested [4]. Heavy metals are a diverse set of highly reactive compounds that can function as cofactors in physiological processes or as toxins [5]. Some metals are necessary for human physiology at all stages of life, but practically all of them can be harmful at certain doses, depending on the chemical form, exposure route, frequency, and duration [1].

Several PTEs, such as lead (Pb), arsenic (As), and chromium (Cr), are known to be harmful to kidneys, lungs, and the nervous system, among other organs and systems. Exposure to these metals can lead to a variety of health problems, including respiratory issues, cancer, anemia, depression, reproductive problems, immune disorders, cardiovascular, metabolic, and renal diseases, and so on [1,3,6]. Thyroid function, reproduction, and metabolism can all be affected by a variety of metals with endocrine system diseases [1,7]. Metabolic diseases are caused by a lack of some important metals, such as Cr, or the accumulation of poisonous metals, such as Pb and cadmium (Cd). Obesity may be associated with Pb and Cd, according to some research. Metals such as zinc (Zn) and Cr, on the other hand, are important trace elements for lipid, carbohydrate, and protein metabolism, and they promote insulin activity in particular [4,8]. Some toxic metals contribute to obesity by being replaced with essential metals including Zn, Cr, copper (Cu), iron (Fe), and magnesium (Mg), which can impact energy generation, glucose tolerance, and other metabolic processes [5].

PTEs have been shown to have an impact on the development of obesity in children in the United States, according to research [9]. Biological monitoring is a well-established method for assessing people's exposure to contaminants in the environment. Metal concentrations in urine are a sign of continuous or cumulative metal toxicity exposure [10]. The use of urine matrix for children and adolescents is a suitable method for biological monitoring since it is non-invasive [11,12].

Many studies have been carried out to look at the association between metal exposure and health, such as Shao et al.'s research, which investigated urine levels of metals in American children. Obesity was found to have a strong positive relationship with barium metal exposure, as well as a negative relationship with cobalt, lead, and cadmium [4]. The findings of Wang et al.'s study revealed that there is a strong association between blood Pb levels and BMI in women [13]. There was no association between blood Pb and As levels in the urine of women of reproductive age and body weight status in the Ronco et al. study [14].

The findings of Earle et al.'s study indicated that BMI and Cr metal metabolism in controls (Healthy), but did not influence persons with diabetes [15]. The findings of a study by Dawood et al., revealed that people with type 2 diabetes have lower levels of Cr than healthy people and that there is a significant relationship between Cr levels and BMI [16]. Anyakudo et al. found that dietary Zn supplementation alters BMI and fat profile in obese diabetic rats [17]. These findings suggest that metals can have an impact on anthropometric parameters and obesity, and consequently on health. Only a few research have been undertaken in Iran to look at the association between environmental contaminants and children's and adolescents' health [[18], [19], [20], [21]]. Due to the significance of heavy metal exposure in children and adolescents, as well as the demographic and anthropogenic traits that emerge and are expressed in daily life, education, family life, and the social system that occurs in all contemporary countries, there is a need for good management in limiting exposure that is utilized globally. The purpose of this study was to see if there was an association between urine As, Pb, Cr, and Zn concentrations and anthropometric and demographic data among children and adolescents in Kerman, Iran.

## 2 Materials and methods

### 2.1 Study population

The current cross-sectional study was conducted in 2020 in Kerman, Iran, on 106 children and adolescents aged 6 to 18. Children and adolescents referred to one of Kerman city hospital's laboratories were randomly selected. In this study, a questionnaire was employed with an informed permission section that was obtained by gaining written agreement from parents, children, and adolescents, and demographic information and physical activity of participants were filled out by parents in the questionnaire [22,23].

### 2.2 Physical examinations

The waist circumference (WC) was measured with a meter. The individuals' height and weight were then measured. The meter was used to measure the height (cm), and the weight (kg) was measured on a digital scale [20]. Then, using WHO AnthroPlus software, BMI was computed by entering information by gender, including age, height, and weight [19].

### 2.3 Urine sample analysis

Previous research has detailed the process for assessing metals in urine samples [22]. In summary, urine samples were obtained from the participants to determine As, Pb, Cr, and Zn concentrations (μg/L) as well as urinary creatinine. Until the day of the experiment, samples were kept in a falcon tube closed at −20 °C. The creatinine in urine was determined using the Jaffe technique and a Hitachi 704 autoanalyzer [24]. The metal content of frozen urine samples was determined after they were melted at room temperature. After that, each 5 mL of urine sample was mixed with 2 mL of 65% nitric acid (HNO_3_, Merck). The samples were then put into an induced coupled plasma mass spectrometry (ICP/MS) instrument [22]. Table 1 displays the ICP/MS device characteristics.

**Table 1:** Characterization of ICP/MS.

Parameters	Value/type
RF generator power	1200 W
RF frequency	Resonance frequency: 24 MHz
Plasma, auxiliary, and nebulizer gas	Argon
Plasma gas flow rate	12.2 (L/min)
Auxiliary gas flow rate	0.8 (L/min)
Nebulizer gas flow rate	0.8 (L/min)
Sample uptake time	260 total (S)
Measurement replicate	3
Type of detector Solid-state	charge-coupled device (CCD)
Type of spray chamber cyclonic	Modified Lichte
Relative Standard Deviation (RSD)	As (11.17%), Pb (10.44%), Cr (2.30%), Zn (9.45%)
Limit of Detection (LOD) [ppb]	As (0.007), Pb (0.019), Cr (0.088), Zn (0.088)
Limit of Quantification (LOQ) [ppb]	As (0.022), Pb (0.062), Cr (0.294), Zn (0.292)

### 2.4 Statistical analysis

After collecting data, which included filling out a questionnaire, measuring the participants' anthropometric parameters, and measuring arsenic, lead, chromium, and zinc in urine samples, first, the normality of the metal variables was checked with the Kolmogorov-Smirnov test, so that the skewness and kurtosis, and the results showed that the variables were normal, then the descriptive and analytical data were statistically analyzed by such as the independent *t*-test (Table 2), univariate regression (in Table 4, the univariate regression was run one by one from demographic and anthropometric factors, and in Table 5, the crude and adjusted univariate regression was run), and correlation tests, using SPSS statistical software. The significance level in all tests is considered 95%.

**Table 2:** Geometric mean urinary As, Pb, Cr, and Zn metal concentrations with and without creatinine modulation, and mean age, creatinine level, and anthropometric parameters in the study population.

Analytes	Total	Girls		p-value df (f)	Boys		p-value df (f)
		6–11 years	12–18 years		6–11 years	12–18 years	
		SD (μg/L) ± Geometric mean			SD (μg/L) ±Geometric mean		
**As**	27.07 ± 20.68	25.56 ± 22.46	33.97 ± 21.39	0.248 1 (1.37)	21.52 ± 10.89	28.65 ± 21.98	0.019* 1 (5.80)
**Pb**	13.64 ± 13.05	10.13 ± 11.34	14.74 ± 10.88	0.133 1 (2.33)	13.47 ± 13.51	16.90 ± 14.57	0.339 1 (0.93)
**Cr**	0.74 ± 0.14	0.78 ± 0.10	0.74 ± 0.17	0.615 1 (0.25)	0.74 ± 0.15	0.71 ± 0.14	0.482 1 (0.50)
**Zn**	239.83 ± 165.00	222.81 ± 156.07	274.33 ± 192.71	0.156 1 (2.07)	210.22 ± 162.73	255.93 ± 154.57	0.321 1 (1.00)
	Geometric mean ± SD (μg/creatinine)				Geometric mean ± SD (μg/creatinine)		
**As**	38.72 ± 39.30	38.69 ± 40.90	48.60 ± 42.13	0.459 1 (0.55)	32.71 ± 17.27	37.54 ± 44.95	0.095 1 (2.88)
**Pb**	19.58 ± 22.91	15.33 ± 19.25	21.08 ± 20.99	0.229 1 (1.48)	20.82 ± 26.47	22.15 ± 24.33	0.848 1 (0.03)
**Cr**	1.06 ± 0.28	1.18 ± 0.15	1.07 ± 0.42	0.427 1 (0.64)	1.14 ± 0.24	0.93 ± 0.24	0.003* 1 (9.65)
**Zn**	344.72 ± 288.16	337.24 ± 286.38	398.10 ± 302.38	0.306 1 (1.07)	322.75 ± 284.48	335.33 ± 289.95	0.797 1 (0.06)
**Creatinine and Anthropometric Variables**		SD ± mean					
**BMI (kg/m**^**2**^**)**	23.21 ± 6.78	24.18 ± 7.76	25.25 ± 7.43	0.628 1 (0.23)	21.68 ± 5.90	24.99 ± 5.85	0.044* 1 (4.23)
**BMI z-score**	1.72 ± 2.15	2.46 ± 2.41	0.95 ± 1.83	0.020* 1 (5.76)	2.15 ± 2.57	1.27 ± 1.55	0.116 1 (2.55)
**WC (cm)**	67.76 ± 12.31	64.03 ± 10.70	72.52 ± 9.62	0.006* 1 (8.32)	61.00 ± 11.13	75.76 ± 11.44	<0.0001*
**Creatinine (μg/mL)**	0.69 ± 0.15	0.66 ± 0.09	0.71 ± 0.16	0.180 1 (1.85)	0.66 ± 0.09	0.79 ± 0.19	0.007*

*p-value is significant at the 0.05 level.

**Table 3:** Distribution of demographic information by sex and age (group).

Variables	Total	Girls		Boys	
		6–11 years	12–18 years	6–11 years	12–18 years
	n (%)			n (%)	
**n (%)**	106 (100)	29 (27.4)	21 (19.8)	22 (20.8)	34 (32.1)
**Father education**					
Illiterate	19 (17.9)	4 (18.2)	6 (17.6)	4 (18.2)	8 (23.5)
Non-academic	73 (68.9)	15 (68.2)	23 (67.6)	16 (72.2)	22 (64.7)
Academic	14 (13.2)	3 (13.6)	5 (14.7)	2 (9.1)	4 (11.8)
**Mother education**					
Illiterate	18 (17.0)	5 (17.2)	3 (14.3)	4 (18.2)	6 (17.6)
Non-academic	67 (63.2)	17 (58.6)	12 (57.1)	15 (68.2)	23 (67.6)
Academic	21 (19.8)	7 (24.1)	6 (28.6)	3 (13.6)	5 (14.7)
**Household income (US$/month)**					
599 ≥	66 (62.3)	19 (65.5)	12 (57.1)	12 (54.5)	23 (67.6)
600≤	40 (37.7)	1. (34.5)	9 (42.9)	10 (45.5)	11 (32.4)
**Physical activity (day)**^a^					
Low	27 (25.5)	7 (24.1)	6 (28.6)	8 (36.4)	6 (17.6)
Moderate	45 (42.5)	15 (51.7)	12 (57.1)	6 (27.3)	18 (32.1)
High	34 (32.1)	7 (24.1)	3 (14.3)	8 (36.4)	24 (42.9)

a Physical Activity: high = more than 30 min, moderate = 5–30 min, low = less than 5 min.

**Table 4:** Association between demographic and anthropometric factors by sex and age (group) with the concentration of urinary analytes (μg/creatinine).

Variable	As (μg/creatinine)					Pb (μg/creatinine)					Cr (μg/creatinine)					Zn (μg/creatinine)				
	Total	Girls		Boys		Total	Girls		Boys		Total	Girls		Boys		Total	Girls		Boys	
		6–11 years	12–18 years	6–11 years	12–18 years		6–11 years	12–18 years	6–11 years	12–18 years		6–11 years	12–18 years	6–11 years	12–18 years		6–11 years	12–18 years	6–11 years	12–18 years
	β	β	β	β	β	β	β	β	β	β	β	β	β	β	β	β	β	β	β	β
Father education																				
Illiterate	−6.35	−53.52*	−5.83	3.71	34.24	−2.26	−3.96	−4.11	−3.54	−1.47	0.21	0.11	0.50	0.19	0.21	57.02	−170.81	−5.04	225.86	238.34
Non-academic	−24.52*	−35.04	−28.04	0.67	−17.40	−10.56	−3.43	−23.19*	0.69	−18.51	0.16	0.18	0.32	0.14	0.09	−98.77	−109.22	−235.99	154.82	−107.65
Academic	Ref	–	–	–	–	–	–	–	–	–	–	–	–	–	–	–	–	–	–	–
Mother education																				
Illiterate	4.43	−25.13	−0.75	24.31*	37.48	5.31	−6.98	4.46	23.45	10.01	0.18	−0.09	0.61*	0.01	0.32	84.91	−67.47	62.33	362.16	208.01
Non-academic	−4.65	4.78	−29.22	9.79	9.93	−1.52	−6.00	−15.26	18.29	1.61	0.07	−0.05	0.19	0.07	0.14	−44.20	−3.26	117.55	205.35	74.80
Academic	Ref	–	–	–	–	–	–	–	–	–	–	–	–	–	–	–	–	–	–	–
Household income																				
(US$/month)																				
599 ≥	0.84	−7.92	−7.02	6.14	7.45	−4.09	−4.14	−1.59	−1.96	−6.97	0.09	0.07	0.20	−0.02	0.15	52.57	106.55	−41.43	183.03	−15.74
600≤	Ref	–	–	–	–	–	–	–	–	–	–	–	–	–	–	–	–	–	–	–
Physical activity (day)a																				
Low	3.24	−14.86	−0.75	13.00	5.40	7.95	5.20	9.14	20.21	−1.27	0.02	−0.14	−0.15	−0.09	0.07	36.26	−241.80	190.45	221.16	−62.77
Moderate	11.19	−4.85	−1.73	2.57	24.73	7.32	8.62	2.41	6.73	14.98	0.01	−0.004	−0.39	−0.06	0.10	107.11	−17.43	176.25	27.72	184.90
High	Ref	–	–	–	–	–	–	–	–	–	–	–	–	–	–	–	–	–	–	–
WC (cm)	0.32	0.54	−0.13	0.40	−0.27	0.11	−0.05	−0.19	−0.24	0.34	−0.01	−0.01	−0.01	0.01	0.01	1.05	3.16	2.98	−1.79	−1.26
BMI (kg/m2)	3.71	2.62	7.71	0.90	5.85	1.39**	0.58	2.04**	0.40	2.82**	−0.01	0.01	−0.01	0.01	0.01	22.99**	22.37**	24.65**	6.20	34.47**
BMI z-score	8.35**	8.27*	17.11**	1.42	21.26**	3.08*	1.96	6.64*	0.60	10.43**	0.02	0.01	−0.01	0.01	0.03	53.48**	62.27*	86.69*	11.31	128.14**

*p-value ≤0.05, ** p-value ≤0.01.

**Table 5:** Association between BMI, BMI z-score, and WC factors with the concentration of urinary analytes (μg/creatinine).

Variable	As (μg/creatinine)		Pb (μg/creatinine)		Cr (μg/creatinine)		Zn (μg/creatinine)	
	β	p-value f (df)	β	p-value f (df)	β	p-value f (df)	β	p-value f (df)
BMI (kg/m^2^)								
**Model 1**	3.71	<0.001 74.22 (1)	1.38	<0.001 2 1.47 (1)	−0.01	0.836 0.04 (1)	22.99	<0.001 44.03 (1)
**Model 2**	3.72	<0.001 71.46 (1)	1.44	<0.001 23.90 (1)	0.01	0.803 0.06 (1)	23.17	<0.001 43.35 (1)
BMI z-score								
**Model 1**	8.35	<0.001 28.28 (1)	3.08	0.002 9.78 (1)	0.02	0.238 1.39 (1)	53.49	<0.001 20.26 (1)
**Model 2**	9.90	<.001 38.72 (1)	3.92	<0.001 15.54 (1)	−0.01	0.951 0.01 (1)	60.65	<0.001 24.27 (1)
WC (cm)								
**Model 1**	0.32	0.299 1.08 (1)	0.11	0.550 0.36 (1)	−0.01	0.250 1.33 (1)	1.05	0.644 0.21 (1)
**Model 2**	0.21	0.584 0.30 (1)	−0.04	0.840 0.04 (1)	0.01	0.149 2.08 (1)	0.09	0.946 0.01 (1)

## 3 Results

Boys aged 12–18 years had greater mean As (μg/L) than boys aged 6–11 years (p = 0.019), while this difference was not significant in girls. The mean BMI in boys aged 12–18 years was higher than in boys aged 6–11 years (p = 0.044), while it was not significant in girls. The mean BMI z-score was greater in 6–11-year-old girls than in 12–18-year-old girls (p = 0.020), while it was not significant in boys. Adolescent girls and boys aged 12–18 years had a greater mean WC (cm) than children aged 6–11 years (p = 0.006). The mean urine creatinine in boys aged 12–18 years was greater than in boys aged 6–11 years (p = 0.007), even though this difference was not significant in girls (Table 2).

The majority of the study population had non-academic father and mother education (68.9% and 63.2% respectively), and 62.3% of the study population had a minimum income of (US$/month) 599. On the other hand, 42.5% of the children and adolescents in the study had moderate physical activity (day) and 25.5% had low physical activity (day). These proportions were similar in the age distribution (6–11 and 12–18 years) sex (boy and girl) (Table 3).

The As (all ages) in children with non-academic father education was 24.52 times (β = −24.31) lower than the children with academic father education. In the entire population, as well as in girls 6–11 and 12–18 years and boys 12–18 years, the association between As and BMI z-score was significant. Pb was 23.19 times lower in girls aged 12–18 years with a non-academic father's education than in girls with an academic father. In addition, in girls aged 12–18 years and boys aged 12–18 years, the association between Pb and BMI z-score and BMI in the entire population was significant. The findings also revealed that the average Cr in girls aged 12–18 years with an illiterate mother was 0.61 times greater than in girls with an academic mother. In addition, in girls 6–11 and 12–18 years and boys 12–18 years, the association between Zn and BMI and BMI z-score was significant in the entire group (Table 4).

Table 5 shows the association between BMI, BMI z-score, and WC variables and urine analytes concentrations (μg/creatinine) in two raw models, the crude model (Model 1) and adjusted model (Model 2, model with the effects of age, gender, and physical activity). Furthermore, in both models, the association between As, Pb, and Zn with BMI (kg/m^2^) and BMI z-score was positive and significant (p < 0.05). The scatter plots of the response (BMI and WC) and variables (As, Pb, Cr, and Zn) are shown in Fig. S1 (in the supplementary file).

Table 6 indicates the metal correlations. The Pearson correlation coefficient demonstrates that the association between Pb–As (r = 0.60), As–Zn (r = 0.74), and Pb–Zn (r = 0.56) is significant (p < 0.001). The findings demonstrated a causal relationship between an increase in some metals' excretion and an increase in other metals' excretion.

**Table 6:** Correlation between metals excretion.

Variables	The correlation coefficient (*p*-value)			
	As (μg/creatinine)	Pb (μg/creatinine)	Cr (μg/creatinine)	Zn (μg/creatinine)
As (μg/creatinine)	–	0.60**	0.11	0.74**
Pb (μg/creatinine)	0.60**	–	0.06	0.56**
Cr (μg/creatinine)	0.11	0.06	–	0.10
Zn (μg/creatinine)	0.74**	0.56**	0.10	–

* p-value ≤0.05, ** p-value ≤0.01.

## 4 Discussion

Children's and adolescents' characteristics, such as age, gender, and BMI, as well as parental socioeconomic position, such as income and education, may influence the rate of heavy metal exposure due to their impact on lifestyle [1]. The results of this study, which focused on the association between demographic characteristics and the concentration of heavy metals in urine, show that parental literacy has an impact on the rate of heavy metal exposure. The concentration of As in adolescent boys aged 12–18 years was greater in this research than in children aged 6 to 11. Differences in food habits or intestinal permeability at various ages might be the cause [25]. As indicated in the results, the accumulation of heavy metals in body tissues grows with age, however the body's metabolism increases, and therefore the rate of heavy metal excretion in urine is greater in those who are older [26]. Comparable findings were found in similar research on pregnant women, indicating that when family income increases, the concentration of heavy metals in the urine rises as well, indicating a positive relationship between income and heavy metals in urine [27]. On the other hand, income can have a beneficial effect on an individual's level of education, such that as the education level grows, the concentration of heavy metals in urine decreases, which may be due to the influence of various cultural, social, and economic variables. As a result, there is a noticeable difference in urinary metal concentrations in persons living in developing and developed societies [28,29].

Physical activity is one of the elements that influence obesity. The participants in this research averaged 5–30 min of physical activity each day, which included doing sports activities, school activities, and playing with friends. Physical activity is undeniably beneficial throughout one's life. Physical exercise in children is crucial for healthy growth and development as well as maintaining energy balance, as well as for enhancing social contacts and well-being [[30], [31], [32]].

The concentration of pollutants in blood and urine, in general, shows how much individuals are exposed to them. When pollutants enter the body, they are first stored in adipose tissue before slowly making their way into the circulatory system via lipolysis. As a result, as the rate of fat lipolysis increases, their serum concentration rises. Physical exercise can lower the concentration of pollutants in the body in a variety of ways. For example, it can boost metabolism and the activity of liver enzymes, sweating, and bile secretions, all of which contribute to higher chemical excretion [[33], [34], [35]]. Physical activity, on the other hand, can increase exposure to pollutants in polluted environments because increased respiration when exercising increases inhalation of pollutants, and because breathing is done more through the mouth, rather than the nose, which naturally filters and adsorbs particles, more particles enter the body. Additionally, increased physical activity increases the speed of air in the respiratory system as well as the penetration of gaseous contaminants as metals attached to particles into the respiratory tract's depths [36]. Children and adolescents, on the other hand, are more sensitive to pollutants due to their greater metabolic rate and level of physical activity, increased respiration, and expanding organs [37,38].

Urinary As and BMI were shown to have a significant association in this study. Heavy metals like As and Pb are endocrine disruptors that can cause insulin resistance and affect glucose metabolism [39]. Arsenic exposure has also been associated with diabetes in studies [40]. Arsenic can raise blood glucose levels via altering skeletal muscle and liver metabolic processes, as well as boosting free fatty acids (FFAs) synthesis and causing insulin resistance through dyslipidemia. Arsenic can also cause oxidative stress, leading glucose metabolism to be disrupted. Furthermore, mitochondrial failure leads to anaerobic metabolism and the formation of reactive oxygen species (ROS) [41,42].

Although obesity is a long-term cause of excessive calorie consumption, research has revealed that gut microbiota influences a variety of physiological activities, including metabolic processing, energy generation, and immune system development. Obesity can influence energy absorption by changing the type or composition of gut microbiota [43,44]. Arsenic has been shown to affect intestinal microbiota in studies [45], indicating that it might cause metabolic alterations and obesity. Arsenic accumulation in body fat, on the other hand, disrupts metabolic pathways and lowers the expression of protein droplet-covering protein (perilipin PLIN1) [41]. Bulka et al. found a negative association between urine As levels and BMI and WC in their investigation [46]. The findings of research by Stahr et al. revealed an association between salivary As levels and obesity [47]. The findings of research by Castriota et al. revealed that As exposure enhanced the incidence of type 2 diabetes [48].

Not all metals are harmful to the body in the same way. Some metals, such as Zn and Cr, are required for the body's proper physiological function at particular levels, whereas others, such as As and Pb, are superfluous for the body and have no recognized physiological role. However, depending on the level of exposure to both metal groups, they may have negative health impacts [49]. Lead is a common environmental toxin that has been associated with a variety of physiological, biochemical, and behavioral problems [5]. Lead poisoning is caused by the metal's affinity for proteins, which interferes with the metabolism of other physiologically essential metals including calcium, iron, and zinc, which function as cofactors in many enzymatic activities and alter the oxidant/antioxidant balance. As a result, it appears to disrupt several enzyme systems, each of which might have a different impact on endocrine function. Lead interferes with the generation, release, biological function, and metabolism of associated hormones, affecting growth, thyroid, and hormone homeostasis, according to studies [50]. Lead, on the other hand, has a high accumulation rate in the body and can accumulate in adipose tissue, bones, and kidneys over time, so as life expectancy and weight gain increase, the amount of Pb in the body increases as well [51,52], indicating a positive association between urinary Pb levels and BMI in the results of the current study. Lin et al.'s findings similarly demonstrate a positive association between urine Pb concentration and BMI Z-Score, which is similar to the findings of this study [53]. In the Shao research, also, there was an association between urinary Pb and obesity in children [4].

Although most hazardous metals have many oxidation states, they can have a variety of impacts on people [54]. By inhibiting the oxidative phosphorylation process, oxygen-activated species can disrupt the normal metabolic activity of mitochondria and the production of energy in the form of Adenosine Triphosphate (ATP). Reduced ATP levels, combined with decreased efficiency of the triglyceride cycle due to inhibition of enzymes like aconitase, cause the liver to direct metabolites to lipogenesis (the storage of fatty acids in the form of triglycerides) [55], which causes the liver to direct metabolites to lipogenesis (the storage of fatty acids in the form of triglycerides). Toxic metals, such as Pb, increase energy storage, which can contribute to obesity. It's also important to remember that people might be exposed to a variety of metals, each of which can have an impact on BMI [56]. Several variables influence the body's vulnerability to hazardous metals. The interplay between the toxicity of essential and harmful elements, which differs from the metabolic state of trace elements, is one of them. One of the harmful pathways might be a disruption in essential element equilibrium. This relationship might be the consequence of interactions during absorption, metabolism, and excretion, or it could be the result of simultaneous exposure to distinct elements [57]. Metal accumulation in the body can occur as a result of metal exposure, as well as variances in behavioral traits, genetics, antioxidant status, availability of protective substances, and other unknown variables that alter the process of metal absorption or distribution in the body [58]. The current study's findings also reveal an association between the metals As, Pb, and Zn. This association might be owing to the presence of a common source via which these metals enter the body at the same time, resulting in an increase in their concentration in the urine [59]. The presence of some heavy metals, on the other hand, will enhance the absorption and excretion of other heavy metals, indicating a relationship between them [60].

Chromium can disrupt metabolically and obesity through a variety of processes, including a fat breakdown and increased lipid profile variables, so a portion of it will be spent on these reactions, reducing its quantity in the urine [61]. In contrast to As and Pb, Cr has a neutralizing function in insulin resistance, making it a glucose-tolerant factor. Chromium may also be an effective factor in the regulation of obesity and diabetes [62]. Studies in mice have demonstrated that as rats acquire weight via feeding, Cr accumulation in adipose tissue diminishes [63]. However, the evidence on Cr status in obese people is insufficient and inconsistent [64]. A study by Son et al. discovered an association between Cr levels and metabolic syndrome [65]. According to studies, Cr bound to chromodoline oligopeptide increases insulin receptor tyrosine kinase activity while inhibiting phosphotyrosine phosphatase activity, increasing the intensity of intracellular insulin signals and thus increasing the effect of insulin on lipid, carbohydrate, and protein metabolism [66]. There was no significant association between urine chromium concentration and BMI in this investigation.

Obesity and overweightness frequently conceal other deficiencies, such as vitamin and mineral deficiencies, which are most likely caused by a poor-quality diet that lacks the micronutrients required to satisfy a person's physiological and metabolic demands. The concentration of Zn in urine in this study was lower than in previous investigations, indicating that children and adolescents are Zn deficient. In children and adolescents, there was also a substantial positive association between urine Zn levels and BMI, according to the findings of this study. Zinc is one of the body's necessary trace elements. Zinc may be found in all parts of the body, including tissues, fluids, and secretions, with a total quantity of 2–4 g in the human body. Zinc is required for a variety of biochemical and metabolic activities. Zinc is involved in the energy metabolism pathways of carbohydrates, proteins, and lipids in biochemical activities. Furthermore, the Zn is a major component of many enzymes involved in the pathophysiology of obesity, and because obese people's metabolism differs from that of others, the amount of Zn excreted in urine is higher in these people, resulting in decreased carbohydrate, lipid, and protein metabolism and obesity [67]. Zinc has been associated with obesity and diabetes in recent research, particularly in terms of altered lipid and insulin profiles, oxidative stress, inflammatory processes, insulin resistance, obesity, and serum leptin levels [68]. Zinc poisoning can weaken the immune system and reduce High-Density Lipoprotein (HDL) levels in the blood [69].

The geometric mean urine concentrations of As, Pb, Cr, and Zn were measured in this investigation and are presented in Table 2 (27.07, 13.67, 0.74, 239.83 (μg/L) respectively). Due to variances in exposure, gender, age, and race of the participants, as well as discrepancies in analytical methodologies such as limit of detection (LOD) in various research, comparing metal concentrations in different studies is difficult [19,70]. In comparison to earlier research, the results of the current study (children and adolescents in Kerman, Iran) demonstrate that geometric mean As, Pb, and Cr concentrations are greater, while Zn concentrations are lower [11,25,71,72]. Metal exposure has increased in recent years as a result of advancements in industry, agriculture, consumer products, and technology. Heavy metals have been found in the environment from a variety of sources, including terrestrial, industrial, agricultural, pharmaceutical, domestic wastewater, and atmospheric sources. Point sources of pollution, such as mining, foundry and smelting, and other metal-based industrial processes, are particularly terrible [73].

Groundwater in Kerman is an important source of water for agricultural and drinking reasons, according to research. The quantity of As in Kerman's groundwater is higher than in other areas of Iran (average 545 μg/L). This percentage is significantly greater than the national standard (NSI) and WHO, which is owing to Kerman's position near Iran's central volcanic area, which is rich in arsenic [74,75]. Despite the presence of such heavy metals in mines such as smelting copper, steel, iron, and coal, as well as manufacturers such as rubber and cement near Kerman, environmental contamination with such heavy metals and their entry into the human body is highly susceptible [76].

Lead, on the other hand, is found in vehicle fuels, chip dust, paints, galvanizing, paint manufacture, leather, steel, cooling water corrosion prevention, and soil [77,78]. According to the aforementioned cases, there are numerous differences in the lifestyle of the studied population, body structure and metabolism, social, economic, cultural, and demographic circumstances, regional industries, ecological and geological structures, weather conditions, and other factors that reveal a conflict between urinary heavy metal concentrations obtained in different studies [29].

The findings indicated that there is an interaction between the excretion of arsenic, lead, and zinc, which has greatly increased each other's excretion and may be brought on by the body's metabolic processes in the concurrent excretion of these metals. On the other side, there can be interactions among heavy metals that make it more rising to remove them all simultaneously [79].

## 5 Study limitations and strengths

One of the research's strengths is its evaluation of As, Pb, Cr, and Zn in children and adolescents, as a sensitive and growing group. However, no research has examined the association between As, Pb, Cr, and Zn with demographic and anthropogenic characterizations in Iranian children and adolescents. The study's major weakness is its being cross-sectional, which implies that causal correlations cannot be determined with certainty using it. The data was gathered from the questionnaire as an admitted acknowledgment that the likelihood of error is high and that the existence of this imperfection is inevitable in questionnaire research.

## 6 Conclusion

Arsenic, lead, chromium, and zinc were found in all samples, indicating that children and adolescents are exposed to these metals. Additionally, the findings revealed a significant association between metal exposure and anthropometric and demographic parameters in the vulnerable group of children and adolescents. As a result, it is essential to decrease children's and adolescents' exposure to heavy metals, as well as their consequences on the health of affected pathways. Because the current study was conducted cross-sectionally, more extensive longitudinal studies are needed to evaluate the effects of metals on the health of children and adolescents.

## Ethical approval and consent to participate

All procedures performed in the study involving human participants were following the ethical standards of the institutional and/or national research committee (IR.KMU.REC.1399.392 ethic approval code).

## Author contribution statement

Majid Hashemi: Conceived and designed the experiments, performed the experiments, Analyzed and interpreted the data; Wrote the paper.

Saeed Rajabi; Habibeh Nasab: Conceived and designed the experiments; Performed the experiments; Analyzed and interpreted the data; Contributed reagents, materials, analysis tools or data; Wrote the paper.

Mostafa Eghbalian: Conceived and designed the experiments, Analyzed and interpreted the data, Wrote the paper.

Joanna Suliburska: Contributed reagents, materials, analysis tools or data, Wrote the paper.

## Funding

This research (with project number 99000223 and IR. KMU.REC.1399.392 ethic approval code) was conducted in the Environmental Health Engineering Research Center of Kerman University of Medical Sciences. This research was supported by the Vice-Chancellor for Research and Technology of 10.13039/501100004621Kerman University of Medical Sciences, Iran.

## Data availability statement

Data will be made available on request.

## Consent of publication

Informed consent was obtained from the legal guardian of the children.

## Declaration of interest's statement

The authors declare that they have no known competing financial interests or personal relationships that could have appeared to influence the work reported in this paper.

## Abbreviations

As: Arsenic
ATP: Adenosine Triphosphate
BMI: Body Mass Index
Cd: Cadmium
Cr: Chromium
Cu: Copper
Fe: Iron
FFAs: Free Fatty Acids
HDL: High-Density Lipoprotein
ICP/MS: Induced Coupled Plasma Mass Spectrometry
LOD: Limit of Detection
Mg: Magnesium
Pb: Lead
ROS: Reactive Oxygen Species
WC: Waist Circumference
WHO: World Health Organization
Zn: Zinc
