# Supplementary figures and images for: Demographic and anthropometric characteristics and their effect on the concentration of heavy metals (arsenic, lead, chromium, zinc) in children and adolescents
Source: Heliyon. 2023 Feb 9;9(2):e13621. doi: 10.1016/j.heliyon.2023.e13621 (PMC9950940; doi:10.1016/j.heliyon.2023.e13621)

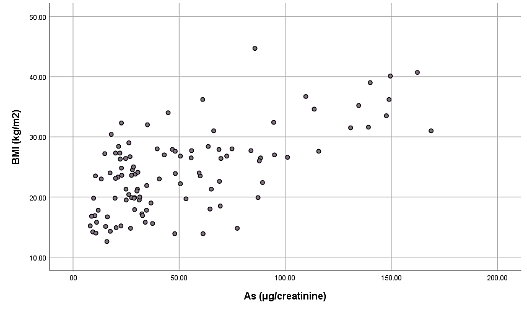

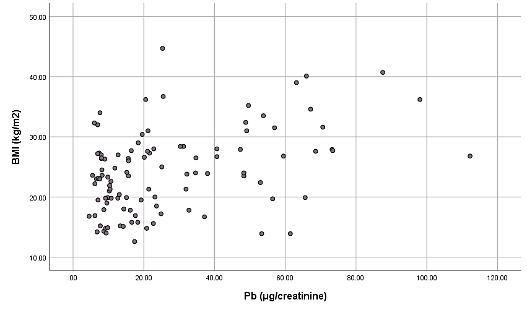

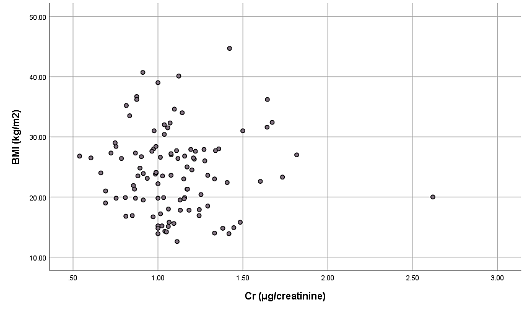

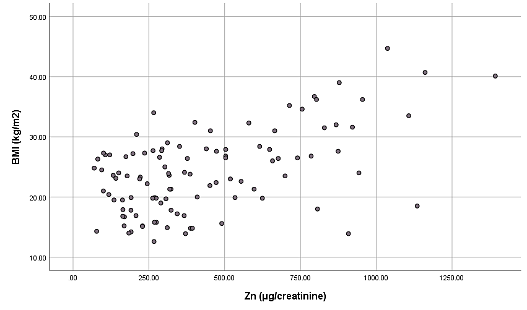


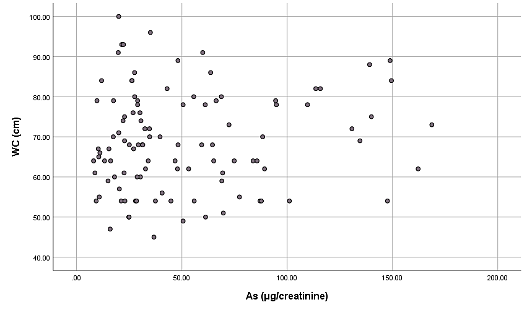

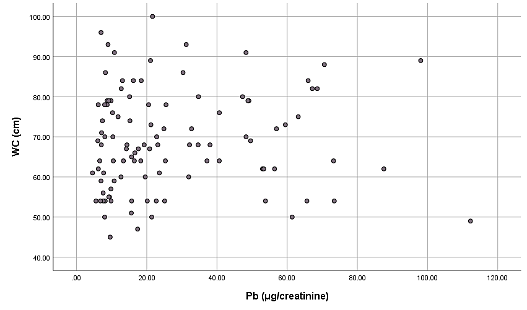

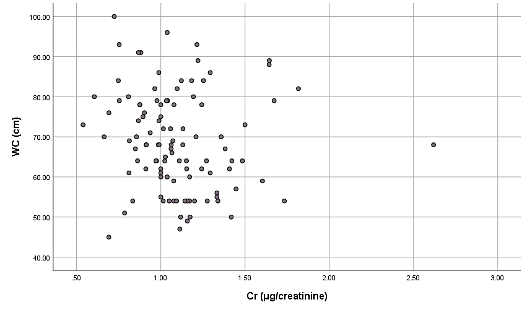

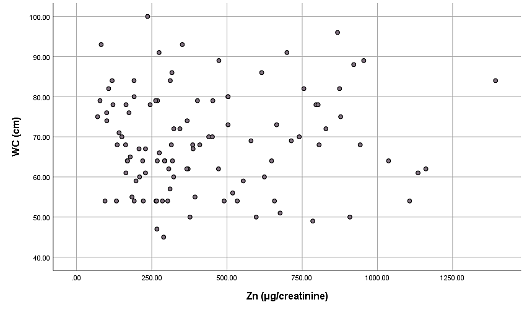


**Fig. S1.** Scatter plots of response (BMI and WC) and variables (As, Pb, Cr, and Zn)

Supplement: Multimedia component 1 [file mmc1.docx]
